# Diagnosis and Treatment of Cardiac Myxoma

**DOI:** 10.7759/cureus.39148

**Published:** 2023-05-17

**Authors:** Nathaniel Manche, Megan K Mercer, Dhiraj Baruah

**Affiliations:** 1 Diagnostic Radiology, Medical University of South Carolina, Charleston, USA

**Keywords:** cardiology cardiac ct and mri, mri cardiac, cardiac neoplasm, cardiac tumor, cardiac myxoma

## Abstract

Cardiac myxoma is the most common primary cardiac neoplasm. It is a benign tumor that typically arises in the left atrium, specifically from the interatrial septum adjacent to the fossa ovalis. We present a case of a 71-year-old male presenting with hematuria that was incidentally found to have a left atrial myxoma on a CT urogram. Follow-up CT and MRI of the heart demonstrated findings compatible with myxoma. Cardiothoracic surgery was consulted, and the patient underwent resection of the left atrial mass, which was confirmed to be a myxoma on pathology.

## Introduction

Primary cardiac tumors are rare with an overall reported incidence of <0.1%, with most of these being benign [1]. The most common benign cardiac tumor is cardiac myxoma, which accounts for nearly 50% of all cases, followed by lipomas and fibromas. Primary cardiac malignancies are rare, with sarcomas being the most frequent. Angiosarcomas are the most common malignant primary cardiac neoplasm in adulthood, and rhabdomyosarcoma is the most common in childhood. In comparison, metastatic disease involving the heart is approximately 40 times more frequent than primary cardiac tumors [1-3]. Diagnosing cardiac myxomas can be difficult as symptoms can vary depending on the location of the tumor [2]. Transthoracic echocardiography can be used for evaluation; however, limitations from poor acoustic windows, operator experience, and body habitus can lead to an incomplete assessment of the heart. MRI is a leading imaging modality used to assess and evaluate cardiac tumors, which helps with not only diagnosis but also prognosis and management [2].

The majority of myxomas arise in adulthood between the fourth and seventh decades of life. A minority of cases are a part of autosomal dominant syndromes such as Carney complex, which is characterized by myxomas, hyperpigmented skin lesions, and extracardiac tumors such as pituitary adenomas, breast fibroadenomas, testicular neoplasms, and melanotic schwannomas [2]. Approximately 20% of cardiac myxomas are asymptomatic. When they do cause symptoms, the most common are primarily cardiac obstructive symptoms related to obstruction of blood flow and constitutional symptoms such as fever, malaise, and weight loss [1,2]. Upon diagnosis, surgical resection is typically indicated [1].

## Case presentation

**Figure 1:**
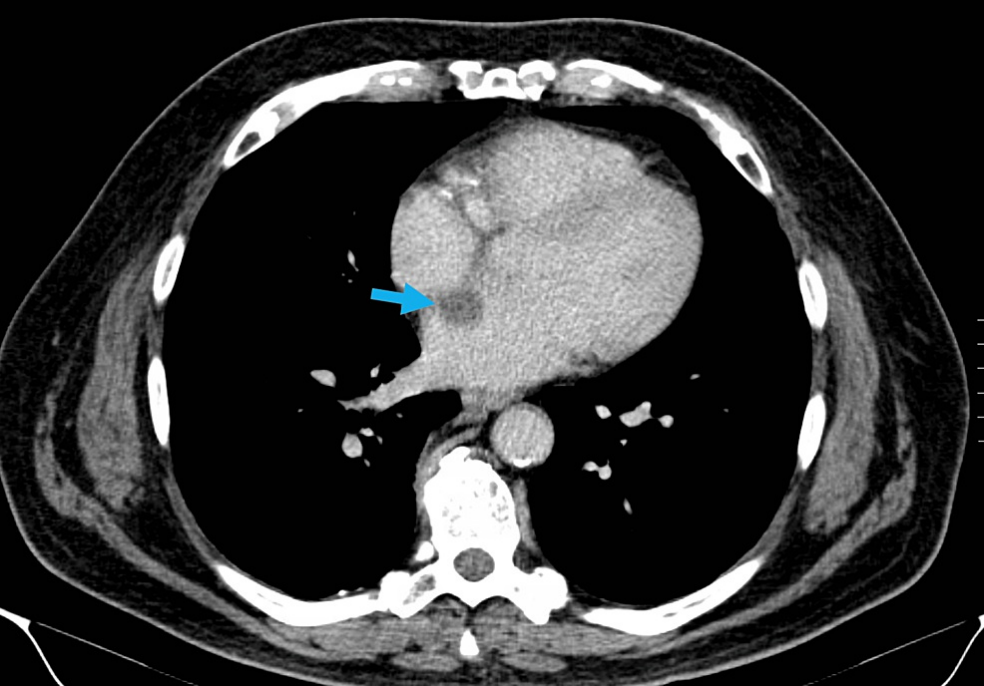
CT urogram showing an incidental partially visualized 2.1 cm round hypodensity extending into the left atrium from the interatrial septum (arrow). CT: computed tomography

We present the case of a 71-year-old male presenting with gross hematuria. His past medical history is extensive, including a stroke within the prior year, hypertension, carotid stenosis, and a transient ischemic attack episode 10 years ago. His surgical history includes a carotid endarterectomy. He is a current smoker with prior alcohol use. Physical examination and laboratory results were unremarkable. The patient initially underwent a CT urogram, which showed a few non-obstructing nephroliths and an incidental partially visualized left atrial mass (Figure 1).

**Figure 2:**
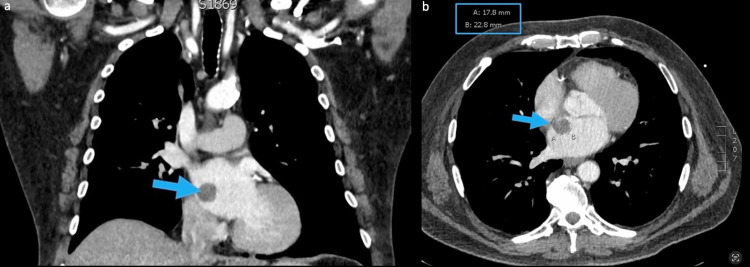
CTA in sagittal (a) and axial (b) views. Pedunculated 2.3 × 1.8 cm hypoattenuating left atrial mass arising from the fossa ovalis of the interatrial septum (arrows). CTA: computed tomography angiography

**Figure 3:**
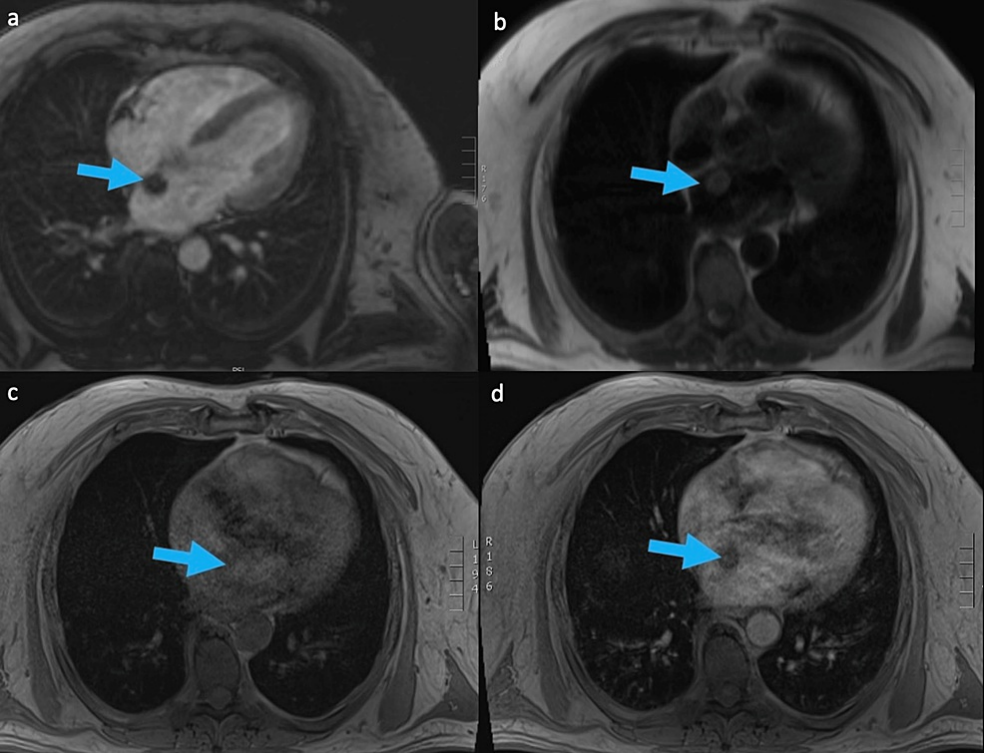
MRI of the heart with and without contrast: (a) four-chamber view, (b) T2 Haste, (c) T1 pre-contrast, and (d) T1 post-contrast (Gd). Pedunculated left atrial mass corresponding with the mass previously seen on CT demonstrating low signal on T1, high signal on T2, and heterogenous enhancement on T1 C+ (Gd) (arrows). MRI: magnetic resonance imaging, CT: computed tomography, Gd: gadolinium

Further cardiac imaging was recommended. The patient then underwent cardiac CT angiography (CTA) protocol, which demonstrated a pedunculated, hypoattenuating 2.3 cm left atrial mass arising near the fossa ovalis of the interatrial septum (Figure 2). No impedance of the mitral valve was visualized. Same-day MRI of the heart was performed, which again demonstrated left atrial mass with low T1 signal, high T2 signal, and heterogenous contrast enhancement (Figure 3).

**Figure 4:**
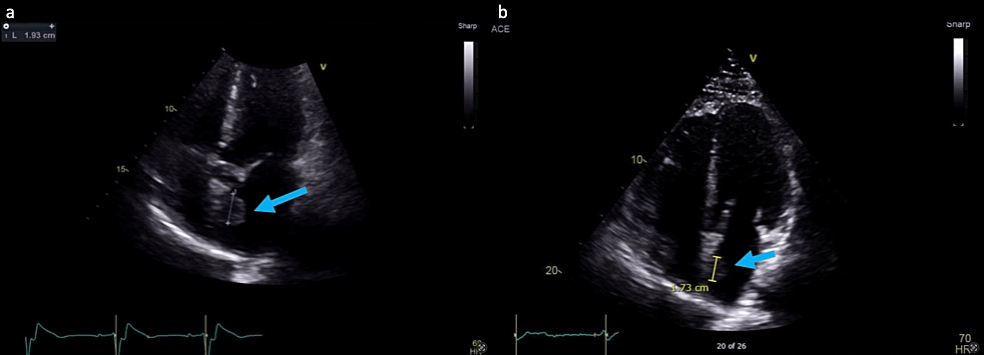
Transthoracic echocardiogram: (a) echocardiogram demonstrating a 1.9 cm left atrial mass and (b) echocardiogram performed three months prior demonstrating the left atrial mass measuring 1.7 cm (arrows).

A transthoracic echocardiogram was also obtained during the workup of the cardiac mass, which showed a non-mobile left atrial pedunculated mass along the interatrial septum measuring 1.9 cm (Figure 4). In comparison, the left atrial mass measured 1.7 cm on a prior transthoracic echocardiogram (Figure 4), which was performed during a stroke workup two months prior to the incidental finding on the CT urogram.

Given the patient’s recent stroke with imaging findings compatible with atrial myxoma, cardiothoracic surgery was consulted for resection. During surgery, a 1.7 cm mass with a clear stalk in the left atrium was removed. Per the pathology report, the resected mass was described as well-circumscribed, round, red-yellow, gelatinous, and hemorrhagic. No gross or histopathological specimen images are available. The microscopic specimens were examined, and pathology confirmed the mass to be a myxoma.

## Discussion

Cardiac myxoma is a benign tumor that constitutes the vast majority of primary cardiac neoplasms. Approximately 90% of myxomas develop in the atria, with 75% arising in the left atrium and 25% arising in the right atrium. Myxomas typically involve the interatrial septum, with most arising immediately adjacent to the fossa ovalis. Macroscopically, myxomas are described as ovoid lesions with smooth, lobular contours and a soft, gelatinous consistency. Microscopically, myxomas are heterogenous with mucinous and cystic areas, hemorrhage, hemosiderin, fibrosis, and calcification [2,3].

Cardiac myxoma can be diagnosed on CT and MRI based on its usual imaging characteristics and location, as described above. On CT, cardiac myxomas are typically spherical or ovoid in shape with lobular or smooth contours. They may be sessile or pedunculated, as demonstrated in our case. Contrast-enhanced CTs will typically show heterogenous enhancement, with the majority demonstrating lower attenuation than the adjacent myocardium. Calcification can be occasionally seen, which is more common in myxomas arising from the right atrium [2].

MRI appearances usually show heterogenous signal intensity, which reflects the heterogeneity of the underlying components of the mass. On T1-weighted imaging, myxomas have low to intermediate signals with increased intensity when there is a hemorrhage. On T2-weighted imaging, intensity can demonstrate low intensity corresponding to fibrous components, or it can demonstrate high intensity corresponding with the extracellular water content of the cystic components. T1-weighted imaging with gadolinium demonstrates heterogenous enhancement, which differentiates it from a thrombus. Increased enhancement may reflect areas of increased vascularity and inflammation, whereas low signal intensity on post-contrast imaging may reflect areas of necrosis [2].

CT and MRI modalities can be superior to transthoracic echocardiogram in the diagnosis and investigation of cardiac masses due to their imaging characteristics. As in our case, a transthoracic echocardiogram may mischaracterize a cardiac mass leading to underdiagnosis. The echocardiogram findings also demonstrate the growth characteristics of a cardiac mass. The mass grew approximately 0.2 cm in the span of two months, which is slower than the documented average growth of 0.49 cm per month [4].

## Conclusions

This case report demonstrates the classic findings and characteristics of a cardiac myxoma through CT and MRI modalities. On CT, the location and appearance of the neoplasm can help determine the diagnosis of a cardiac myxoma, as the vast majority are ovoid and arise near the fossa ovalis along the interatrial septum. MRI can also be used to further evaluate cardiac masses. Specifically, a cardiac myxoma will typically show heterogenous enhancement on a gadolinium contrast study, which differentiates it from a thrombus. The overall heterogenous characteristics on MRI reflect the underlying heterogenous pathology of a myxoma. Increasing enhancement corresponds with increased vascularity and inflammation of a myxoma, while increasing T2 intensity reflects the cystic components. Radiologists should recognize these important imaging characteristics of benign cardiac neoplasms such as myxoma to help with diagnosis, prognosis, and management.
